# Carnivore Management Zones and their Impact on Sheep Farming in Norway

**DOI:** 10.1007/s00267-019-01212-4

**Published:** 2019-10-17

**Authors:** Geir-Harald Strand, Inger Hansen, Auvikki de Boon, Camilla Sandström

**Affiliations:** 1 Department of Survey and Statistics, Norwegian Institute of Bioeconomy Research, NIBIO, Ås, Norway; 2 Department of Natural Resources and Rural Development, Norwegian Institute of Bioeconomy Research, NIBIO, Ås, Norway; 3 Department of Political Science, Umeå University, Umeå, Sweden

**Keywords:** Carnivores, Livestock, Predation, Zoning, Pasture, Sheep

## Abstract

We investigated the impact of Norway’s current zonal carnivore management system for four large carnivore species on sheep farming. Sheep losses increased when the large carnivores were reintroduced, but has declined again after the introduction of the zoning management system. The total number of sheep increased outside, but declined slightly inside the management zones. The total sheep production increased, but sheep farming was still lost as a source of income for many farmers. The use of the grazing resources became more extensive. Losses decreased because sheep were removed from the open outfield pastures and many farmers gave up sheep farming. While wolves expel sheep farming from the outfield grazing areas, small herds can still be kept in fenced enclosures. Bears are in every respect incompatible with sheep farming. Farmers adjust to the seasonal and more predictable behavior of lynx and wolverine, although these species also may cause serious losses when present. The mitigating efforts are costly and lead to reduced animal welfare and lower income for the farmers, although farmers in peri-urban areas increasingly are keeping sheep as an avocation. There is a spillover effect of the zoning strategy in the sense that there is substantial loss of livestock to carnivores outside, but geographically near the management zones. The carnivore management policy used in Norway is a reasonably successful management strategy when the goal is to separate livestock from carnivores and decrease the losses, but the burdens are unequally distributed and farmers inside the management zones are at an economic disadvantage.

## Introduction

Populations of large carnivores are recovering in many parts of Europe, following a long period of decline (Woodroffe 2000; Treves and Karanth 2003; Eriksson 2017). Roughly one-third of the continent is now host to at least one of the five large carnivore species: brown bear (*Ursus arctos*), wolverine (*Gulo gulo*), gray wolf (*Canis lupus*), lynx (*Lynx lynx* and *Lynx pardinus*), and golden eagle (*Aquila chrysaetos*) (Chapron et al. 2014). The preceding decline in the number of large carnivores was, at least partly, caused by the expansion of agriculture and the resulting conflicts between carnivores and livestock (Mattiello et al. 2012). Studies also show strong associations between high human population density and the loss of carnivores from a region (Cardillo et al. 2004; Safi and Pettorelli 2010).

The meticulous development and implementation of successful conservation strategies is perhaps the single most important factor explaining the return of the large carnivores in Europe. An additional and possibly influential factor is the changing modes of agricultural production. Traditional and economically less intensive production methods have been marginalized in favor of an industrialization of the agricultural sector (Martin 2001). The bulk of meat, poultry, and dairy production in most European countries today take place in controlled environments, inaccessible to carnivores and carnivores are no longer a threat to food security. Attitude surveys subsequently show strong support for the current conservation policies regarding large carnivores (Skogen 2001; Kaltenborn and Bjerke 2002; Røskaft et al. 2007; Blekesaune and Rønningen 2010; Eriksson 2017).

The changing social and economic conditions have fostered urbanization followed by rural depopulation. This is part of a common trend across Western Europe (Rey-Benayas et al. 2007; Navarro and Pereira 2015). Farming is abandoned on many smaller farms (now converted to holiday homes), leading to afforestation and creation of suitable habitats for large carnivores. The social effect of the urbanization is that the majority of the population has little or no first-hand knowledge of the rural society or agricultural production. The result is an increasing social distance between the rural culture and the majority of the population.

Livestock production is, due to climatic constraints, important in Norwegian agriculture. Norway is located between 58° and 71° latitude in the Northern Hemisphere. Only 3% of the area is cultivated agricultural land and the majority can only be used for grass production. Grass must be processed by animals and refined into meat or dairy products in order to be used as food for humans.

Norway has short summers and long winters. Farmers therefore harvest the grass produced on the scant agricultural land during the summer and use it as fodder for the animals during the winter. Fortunately, there are also rich, but uncultivated pastures in the Norwegian forests and mountains where the livestock can rummage for food during the summer. Norwegian agriculture therefore distinguishes between “infield” and “outfield” pastures. The “infields” is the cultivated agricultural land and “infield pastures” is pasture on cultivated (and usually fenced) fields. The infields can be used as pasture early in the spring and late in the autumn, but the main function is to cultivate hay that is harvested and stored for use during the winter. The “outfields” is the unmanaged and unfenced pasture in forests, mountains, fens, moors, and heathland. These are exploited by free-roaming livestock during the summer months. The combined use of “infields” and “outfields” constitutes a production system that has been operational for several centuries. Carnivores were exterminated in order to protect livestock on outfield pastures. The reintroduction and subsequent growth of large carnivore populations has led to a revival of old human–carnivore conflicts, this time as a political conflict with strong economic and societal connotations (Eriksson et al. 2015).

Carnivore predation on livestock occurs when predators and livestock are present in the same area. Total losses are often small relative to the total numbers of livestock, but can still constitute a significant proportion of total livestock mortality. Juvenile animals are particularly vulnerable. Losses are highly variable, but can be geographically concentrated, resulting in very-high loss for some herders (Baker et al. 2008). A study of the economic impact of protected large carnivores on sheep farming in Norway at the turn of the century documented considerable losses in some areas (Asheim and Mysterud 2004) and predicted that the losses experienced by sheep farmers could cause sheep-farm decline. Direct losses also increase the conflicts between involved interest groups. The key to successful coexistence requires limiting livestock losses to levels that are acceptable to a majority of the affected community (Dorresteijn et al. 2013).

The Norwegian parliament has sought to establish a compromise between the stakeholders in the human–carnivore conflict. The solution is a political consensus formalized through two parliamentary decisions. The first was decision 337 (13th May 2004) over proposal Innst. S. 174 (2003–2004).The second was decision 687 (17th June 2011) over proposal 163S (2010–2011). These decisions are known to the Norwegian public as the Carnivore Settlements of 2004 and 2011, respectively. The settlements seek to reconcile two goals: continued sustainable livestock production in the outfields and the maintenance of viable carnivore populations.

The main tool developed under the Carnivore Settlements of 2004 and 2011 is a zonal management system (Ministry of Environment 2003; Hansen et al. 2019). Carnivore Management Zones (CMZ) are defined individually for each carnivore species by eight regional carnivore management boards. The total CMZ area for a species must be large enough to sustain a viable carnivore population, where “viable” is a population size defined by the parliament. The target is currently 65 annual litters for lynx, 39 for wolverine, and 13 for bear. The annual target for wolf is four litters by reproducing groups in Norway and two litters by groups that may have part of their territory in Sweden (with each litter in a partially Swedish pack weighted by 0.5).

The CMZs are drawn up independently for each carnivore species, and there is considerable spatial overlap between the zones. The remaining area (not allocated to one or more carnivore species) is considered as prioritized for livestock. The concept “prioritized for livestock” is equivocal. Many areas fall inside the CMZ for some, but not all carnivores. An example is the large areas assigned as CMZ for lynx, but outside the CMZ for the other three carnivore species. Livestock in these areas must be protected against lynx, but brown bear, gray wolf, and wolverine should not be expected here.

A CMZ is not a sanctuary and outfield pasture can also be utilized inside a CMZ, but only provided that sufficient and adequate steps are taken to avoid conflicts with carnivores. Such steps include fencing, shepherding, guard-dogs, and physically moving the livestock to new locations. Lethal population control can be used to regulate the carnivore population inside the CMZs upon reaching the population targets.

The fact that a location is included in a CMZ does not necessarily imply that carnivores are present. There will be regions without carnivores inside the CMZs, and there will be carnivores present outside the CMZs. It is, however, more likely that carnivores are present inside the CMZs, since the protection is stronger there. There are also differences with respect to grants provided for mitigation and compensation schemes inside and outside the CMZs (Hansen et al. 2019). The considerable overlap between CMZs also implies that the impact from carnivores may be higher in certain regions due to carnivore sympatry.

The objective of this paper is to examine the impact of the reintroduction of large carnivores and the creation of a zonal carnivore management system on livestock agriculture, using the current situation in Norway as a case study. We focus in particular on the effect of the zonal management approach on the sheep industry and discuss possible mitigation efforts to alleviate future conflicts.

## Method and Material

Digital maps of the management zones (CMZ) for the four mammalian large carnivores were downloaded from the Norwegian Environment Agency (production date 15th September 2015), converted to a common projection (UTM-33/EUREF89) and merged into a single dataset. Slivers and gaps were removed, geodetic errors were corrected and the boundaries set to match the coastline and national boundary from official topographic datasets at scale 1:50,000 retrieved from the national geospatial infrastructure (Norway Digital). The result was a polygon map where each polygon was represented with four binary [0, 1] variables indicating that the polygon was (1) or was not (0) part of a management zone for the corresponding four large carnivore species.

Organized Outfield Grazing (OOG) is a system introduced in 1970 to improve animal welfare, reduce loss of animals during the grazing season, and increase profitability for the farmers. OOG invited farmers to form local grazing associations (LGA) and cooperate in capacity building with respect to tending and herding animals on outfield pastures. LGAs are entitled to public subsidies and have to report annually on activities, number and loss of livestock, as well as obtained weights. These data are available in a central database known as the Information system for outfield grazing (IBU). The outfield area used by each LGA has been mapped and the geographical information is also available as part of IBU.

Data for all operative Norwegian farms in 1999 and 2017 were retrieved from the register of applications for agricultural subsidies (older data are currently not available). These records were linked to the national farm register in order to retrieve point coordinates for the farmsteads and establish a point dataset with attributes representing the farmland in use and the number of different livestock animals on each farm. A point-in-polygon overlay with the CMZ-map was used to add four binary indicator variables representing the presence or absence of each carnivore management zone at the farmstead. These indicator variables allowed us to stratify the farms according to their location, inside or outside any particular CMZ, or combination of CMZs.

Data on loss of sheep, from the annual reports submitted by the LGAs, were aggregated by county and year from 1970 to 2016. Relative loss was calculated as a percentage of the number of animals that were released in the outfield grazing areas. There was no attempt to identify the cause of loss in this material. In addition to total national figures, two counties were selected to represent two extreme situations. Hordaland, on the Atlantic Coast, is a region with very few large carnivores, no CMZ and relatively minor impact of diseases associated with outfield grazing. Hedmark, on the Swedish border, is also a region with few problems related to disease, but has management zones for all four large carnivore species within the county boundaries and a high impact of gray wolf and brown bear migrating from Sweden (Ministry of Climate and Environment 2016, p. 26). The development in Hordaland and Hedmark were compared using descriptive graphics.

The survey data from the Norwegian area frame survey of land cover and outfield land resources (Strand 2013, Bryn et al. 2018) was post-stratified using the CMZ-map and then used to calculate grazing capacity inside the zones. Data from the LGAs were also post-stratified using the zonal map in order to calculate the current use of the grazing capacity in each zone. Grazing capacity and actual exploitation of the grazing resource was calculated for a number of partly overlapping strata. The unit is “Livestock units” (LSU), a reference unit which facilitates the aggregation of livestock from various species and age. The reference unit used for the calculation of 1 LSU is the grazing equivalent of one adult sheep.

The Norwegian Nature Inspectorate (SNO) examines carcasses of domestic animals found and reported by the farmers, in order to determine the cause of death. The methods and routines used in the field by SNO are described in detail in Skåtan and Lorentzen (2011). Only a fraction of the animals lost and claimed are found and examined by SNO, but the cases reported by SNO can probably be considered as a valid sample of the animals actually killed by carnivores. This proposition is based on the fact that SNO is a national public authority and is present with trained and professionally well calibrated local officers in every part of Norway, and also on the assumption that the likelihood of finding the carcass of a dead animal is independent of location. The material may be biased if carcasses are harder to find in certain regions, or SNO officers have developed dissimilar practices in different regions.

The data created by the examination of carcasses carried out by SNO are available in the database Rovbase (www.rovbase.no) and include location (measured using GPS), the probable cause of death and a remark about how reliable the information is (how certain the SNO officer is about the cause of death). We downloaded the data and used a GIS overlay tool to link the carcass observations to the management zones. The link was used to stratify the carcass observations into two strata: carcass observations inside the CMZ and carcass observations outside the CMZ for the carnivore that killed the prey. We used this stratification to calculate the relative number of sheep killed outside the CMZ for each carnivore species.

We could not get access to any dataset showing the geographical distribution of each of the four large carnivore species. Instead, we used the carcass data from SNO as a proxy. The carcass observations were linked to a 25 × 25 km national statistical grid developed by Statistics Norway (Strand and Bloch 2009) using a GIS intersection tool. We counted the number of years each carnivore species killed one or more domestic animals in each grid cell. The four resulting maps show how frequent, in terms of number of years, domestic animals (including dogs and reindeer) was killed by the carnivores in each grid cell. We consider this map as a simple indicator of the species distribution.

## Results

**Fig. 1:**
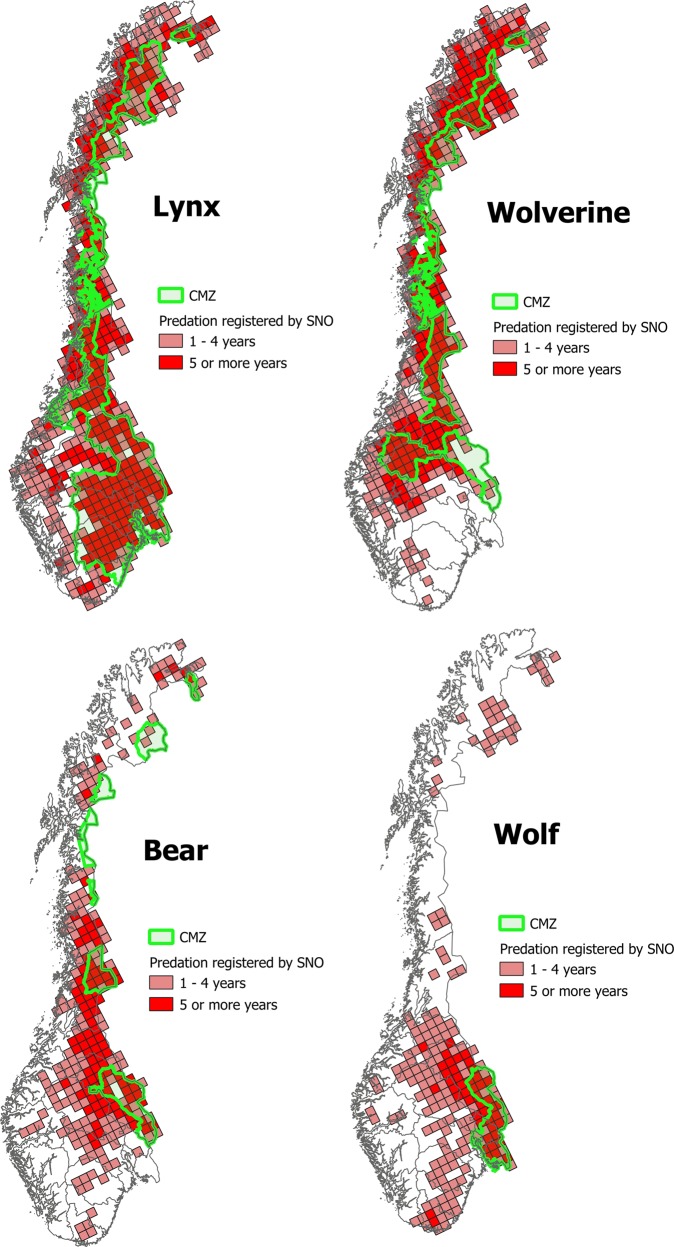
Management zones and estimated population range for brown bear, lynx, gray wolf, and wolverine in Norway. Population range is estimated by counting the number of years domesticated animals (including dogs and reindeer) have been registered by SNO as killed by the carnivore species since 1990 inside 25 × 25 km grid cells

The CMZs in Norway cover ~180,000 km^2^, or 55% of the Norwegian land area. The CMZ for lynx constitutes the largest parts of this area (~149,000 km^2^), often intersecting CMZs for other large carnivore species. The zones for wolverine, brown bear, and gray wolf are smaller. The CMZ for gray wolf covers ~18,000 km^2^ in south-eastern Norway. The four CMZs are shown in Fig. 1(a–d) along with the estimated geographical distribution of the four carnivore species. The number of years when SNO has found carcasses of livestock (including dogs and domesticated reindeer) killed by a particular carnivore species is used as an indicator of presence for that carnivore in the grid cell. The maps differentiate between grid cells where the carnivore species only has killed domestic animals in 1–4 years since 1990 (when the registrations started) and grid cells where the species has killed domestic animals in 5 or more years during the period. This is not an exact species distribution map, but in our opinion a reasonable approximation in the absence of more accurate data.

We notice, however, that the maps underestimate the presence of carnivores arriving in an area after the removal of livestock. An example is wolverine. Wolverine is usually found in the mountains, but has also migrated into forested areas, because the wolverine is attracted by remains of moose (and other prey) killed by the larger carnivores. This migration followed the reintroduction of brown bear and gray wolf in the forest areas. Sheep was already removed from these outfield pastures when the wolverine arrived. They therefore appear incorrectly as white spots on the wolverine distribution map, because no carcass of domestic animals killed by wolverine has been observed in the area. We know that the wolverine is present because the species have been observed by local hunters (pers. comm.).

The success with respect to achieving the targets for viable carnivore populations in Norway is reported by the environmental authorities using the web site https://miljostatus.miljodirektoratet.no/tema/arter/rovdyr-og-rovfugler/. The reports for 2019 show that the population targets were achieved for gray wolf and wolverine, but not for lynx and brown bear. Further discussion regarding the achievement of the population targets is outside the scope of this paper, but can be found in Krange et al. (2016), Swenson et al. (2017), Gervasi et al. (2019) and López-Bao et al. (2019).

**Fig. 2:**
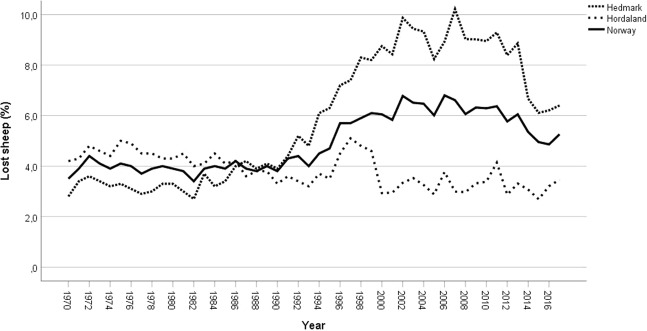
Percentage of sheep grazing in the outfields that are missing after the grazing season. In addition to national figures (Norway) the graph includes figures for two counties: Hordaland on the Atlantic Coast (with few large carnivores) and Hedmark (in the eastern part of the country, bordering Sweden, and with growing carnivore populations since ~1990)

**Fig. 3:**
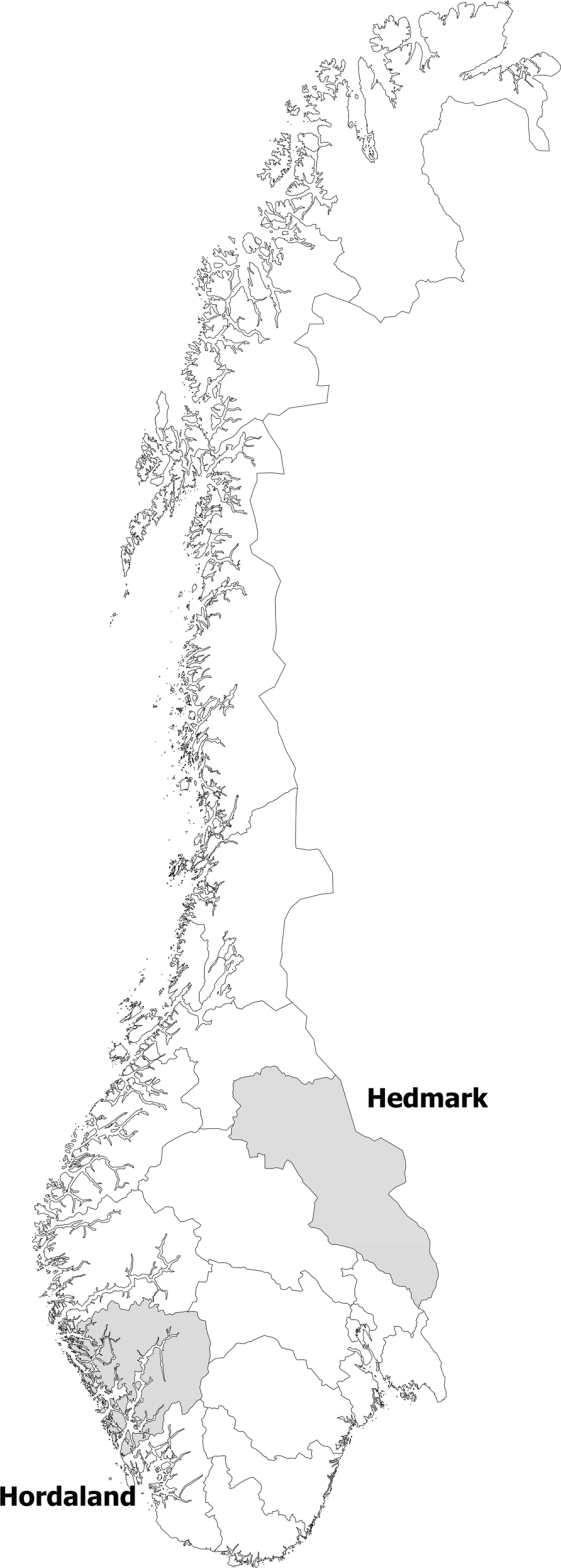
Location of Hordaland (on the west coast) and Hedmark (on the eastern border) counties

Figure 2 shows the loss of sheep (percent lost) on outfield pastures by year during the period 1970–2016. The lines represent the national average together with two selected counties: Hordaland and Hedmark (representing two different environments with respect to carnivores. Figure 3 shows the location of Hordaland and Hedmark). The graph shows reduced loss rates in both counties from 1970 into the early 1980s, when loss rates started to increase in Hedmark but not in Hordaland. The first increase in Hedmark is concurrent with the reintroduction of brown bears. The loss rates furthermore accelerated rapidly in Hedmark after 1990, a development coincidental with the reintroduction of gray wolves in this county. No carnivores have been reintroduced in Hordaland. The losses stabilized in Hedmark in 2004, when the zoning management strategy was implemented, and decreased sharply in Hedmark from 2014. A more detailed explanation of these results is found in the “Discussion” below.

**Fig. 4:**
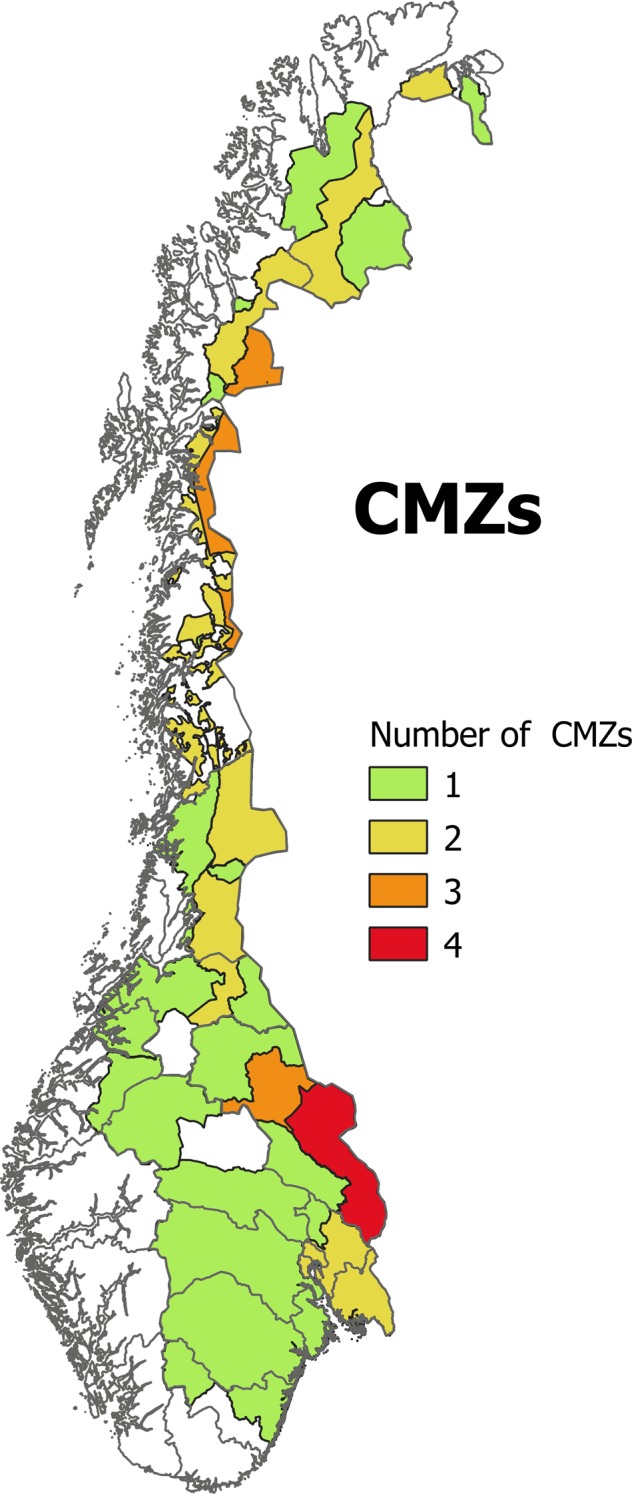
Management zones for bear, lynx, wolf, and wolverine overlap. The map shows the number of carnivore species found in each area. Areas with no carnivore species are prioritized for pasture

The CMZs for the four large carnivore species do to some extent cover the same tracts of land (Fig. 4). Some areas and some farmers are therefore located inside the CMZ for several carnivore species. Provided that the carnivores are present, the situation is most severe in a region covering ~9000 km^2^ in Hedmark, where CMZs for all the four large carnivore species intersect.

The structural change in Norwegian agriculture is described by comparing data from applications for subsidies from 1999 to 2017 (Table 1). These data were stratified according to the CMZs. The table has several sections. The first section (a) shows percent change for the entire country. The second section (b) compares the percent change inside and outside CMZs. There is little difference between these strata, but sheep farming decreased inside the CMZs and increased in areas prioritized for livestock.

**Table 1:** Changes (%) in agricultural activity from 1999 to 2017 for (a) Norway; (b) inside and outside carnivore management zones; (c) among four species management zones; (d) northern and a southern part of the wolf management zone; and (e) management zones for one, two and three to four carnivore species

	Change (%) from 1999 to 2017				
Region	Active farms	Agricultural area	Grassland area	Number of sheep	Number of sheep farmers
(a) Norway	−39.4	−4.0	0.1	4.8	−33.1
(b) Outside carnivore management zones	−40.2	−4.2	−3.3	7.6	−32.8
Inside carnivore management zones	−38.7	−3.9	5.1	−1.3	−33.9
(c) Management zone for bear	−50.1	−6.4	18	−38.5	−48.3
Management zone for wolverine	−43.5	−4.9	−3.9	−5.4	−39.4
Management zone for lynx	−38.8	−4.1	5.5	−1.5	−33.6
Management zone for wolf	−40.1	−4.9	13.5	11.3	−20.6
(d) Management zone for wolf—southern part	−36.3	−4.4	16.7	55.3	−7.9
Management zone for wolf—northern part	−51.6	−6.9	5.7	−42.2	−45.6
(e) Management zones for one carnivore species	−37.5	−3.2	7.0	0.1	−33.8
Management zones for two carnivore species	−38.2	−4.9	−1.9	1.1	−30.2
Management zones for 3 or 4 carnivore species	−51.9	−6.8	4.6	−33.2	−47.3

The third section of Table 1 (c) shows percent change inside the CMZ for each of the four large carnivores. Note that these zones to some extent cover the same areas (Fig. 4). The table shows that the number of sheep and the number of sheep farmers both decreased substantially in the CMZ for brown bear. The number of active farmers also decreased more inside this CMZ than in other zones. The result shows that agriculture in general, and sheep farming in particular, is faced with major challenges in the region designated as management zone for brown bear.

The fourth section of Table 1(d), divides the CMZ for gray wolf into a northern and a southern part of approximately equal size. The stratification follows the administrative boundaries. Family groups of gray wolf are well established in the northern part, and this part also coincides with the CMZ for brown bear. The southern part is outside the CMZ for brown bear, and has few resident gray wolf packs. The number of sheep increased in the southern, but decreased substantially in the northern part of the zone. The reduction in the number of active farmers is also less than the national average in the southern part, but substantially higher than the national average in the northern part.

Finally, the fifth section of Table 1(e) shows the change in areas within CMZs for none, one, two or three to four carnivore species. The table indicates more austere structural changes in terms of reduced number of farmers, agricultural land in use, and number of livestock in areas where CMZs for three or four carnivore species overlap and carnivore sympatry may be present.

The results pertaining to grazing capacity (Table 2) show that the overall exploitation of outfield grazing resources in Norway is ~40% of the available resources. The geographical distribution is uneven. The use in areas outside CMZs is considerably higher (59%) than the use in areas inside the CMZs (26%). The lowest exploitation of available grazing resources is found inside the CMZ for brown bear (6%) and gray wolf (12%).

**Table 2:** Grazing capacity on outfield^a^ pastures in Norway (total) and inside management zones for carnivores

	Livestock units (LSU)^b^		
Region	Pasture capacity (LSU)	Actual use (LSU)	Percent use
Norway (total)	7,492,000	3,008,000	40%
Outside carnivore management zones	3,230,000	1,921,000	59%
Inside carnivore management zones	4,263,000	1,087,000	26%
Management zone for bear	673,000	42,000	6%
Management zone for wolverine	3,515,000	948,000	27%
Management zone for lynx	1,745,000	280,000	16%
Management zone for wolf	379,000	46,000	12%

^a^Outfield pasture is defined as unmanaged and unfenced pasture in forests, mountains, fens, moors, and heathland, where the livestock roam freely

^b^One LiveStock Unit (LSU) is the grazing equivalent of one adult sheep

Table 3 shows statistics based on the location of carcasses of dead sheep. The table includes only carcasses where the SNO officer has identified the cause of death with high certainty as being due to carnivores (as defined in Skåtan and Lorentzen 2011). Lynx was the only carnivore where the majority of the carcasses (65%) caused by the species were found inside the CMZ for the species. Carcasses caused by other large carnivores were mainly found outside the CMZ for the species.

**Table 3:** Carcasses of sheep killed by specified large carnivores from 2000 to 2015 inside and outside the CMZ

	Inside management zone		Outside management zone		Total number
Carnivore species	Number	%	Number	%	
Bear	2730	25.1	8162	74.9	10,892
Lynx	4584	65.0	2472	35.0	7056
Wolverine	3262	31.7	7013	68.3	10,275
Wolf	1192	21.7	4291	78.3	5483

A summary of the main results are

- Increasing sheep losses coincide temporally and geographically with the reintroduction of large carnivores.
- Sheep farming is slowly moving from areas inside CMZs to areas outside CMZs.
- Sheep farming decreased most in areas where several CMZs overlap, and in particular inside the CMZ for brown bear.
- Sheep farming is decreasing in the northern and increasing in the southern part of the CMZ for gray wolf.
- Outfield grazing resources are less exploited inside CMZs than outside CMZs.
- Sheep losses have decreased inside CMZs since the introduction of the zoning policy.
- Carcasses of sheep killed by carnivores are now mainly found near, but outside the CMZs.

## Discussion

Our results show that the zoning strategy is successful in terms of separating livestock from carnivores. This is partly attained by moving livestock from open to fenced pastures inside the CMZs, partly by moving livestock to grazing areas outside the CMZs. Some farmers in areas with high depredation also give up sheep farming entirely. Sheep farming is thus gradually transformed or abandoned in the areas where carnivores are present. The result is less depredation, but also a loss of employment and income, and reduced use of grazing resources. Direct conflicts between livestock and carnivores are reduced inside the CMZs, but the human–carnivore conflicts continue as a result of the societal effects of the changes. There is also an increasing conflict due to spillover effects in the areas surrounding the CMZs.

### Increasing Sheep Losses Coincides with the Reintroduction of Large Carnivores

The time series based on data from IBU shows the long-term development of losses of sheep in the outfields. Around 80% of the livestock using the outfield pastures in Norway are kept by farmers who are members of an LGA. Figure 2 compared losses in the two counties Hedmark and Hordaland. We interpret the difference between Hedmark and Hordaland as the effect of the reintroduction of large carnivores in Hedmark.

As shown in Fig. 2, the relative loss of sheep during the grazing season was fairly similar in Hedmark and Hordaland before the reintroduction of large carnivores in Hedmark. Hedmark actually had relatively lower losses (3%) than Hordaland (>4%). Both counties also showed a positive development with falling losses throughout the initial years. A first change set in around 1982 when the brown bear had returned to Hedmark. Losses now started to increase in Hedmark while losses in Hordaland continued to decline. The reintroduction of the brown bear is the only judicious explication. Farmers in Hedmark were not prepared when the bears returned, had little or no experience with carnivores, and were unable to implement any effective protective measures. From 1990 onward, the losses increased dramatically in Hedmark. These are the years when the gray wolf also returned and the first packs were established in the region. Losses in Hedmark stabilized after the turn of the century. The high losses continued until 2010, but are later substantially reduced. The zoning management strategy was introduced in 2004, and mitigation efforts (in terms of expelling sheep from the outfield pastures) started to take effect a few years later.

The development in Hedmark is closely linked to the reintroduction of the large carnivores. Losses increased when the large carnivores were reintroduced and declined again when the CMZ strategy was implemented and farmers gave up free ranging sheep farming and stopped using local grazing resources. Many farmers abandoned sheep farming altogether. Those that remain keep their sheep inside enclosures with carnivore-repellant fences or transport the sheep to rented summer pastures in regions outside the CMZs for brown bear and gray wolf.

The impact on agriculture is not as pronounced inside CMZs for lynx and wolverine, where the pressure from large carnivores is less severe than in the CMZs for gray wolf and brown bear. Farmers in these areas have organized themselves and adopted techniques to protect the livestock. The techniques include transportation of sheep to regions with less carnivores, intensified guarding, organization of local communities prepared to help muster the sheep, and bring them back from the outfields when carnivore attacks set in, and provision of prepared and fenced infield areas where sheep returning early from the outfields can graze.

Increasing livestock losses when large carnivores are reintroduced in areas with grazing livestock on open pastures is also reported from other countries (Stahl et al. 2001; Kaartinen et al. 2009; Meuret et al. 2017; Widman and Elofsson 2018). Studies show that losses can be geographically concentrated, (Stahl et al. 2002; Scasta et al. 2018; Behmanesh et al. 2019) and national or even regional figures can cover substantial variation between locations. There are few longitudinal studies, however, and our time series provides new insight into the dynamics, since we can follow the development over a period of 50 years and compare regions that are different in terms of carnivore composition.

### Geographical Shift in Sheep Farming

The Norwegian agricultural sector has gone through considerable structural changes since World War II (Forbord et al. 2014). The number of active farmers went from 213,000 in 1949 (Bye et al. 2014) down to 40,000 in 2017. Most of the farmland is still in use, suggesting that production takes place on fewer, larger, and more capital-intensive farms. The greater part of these structural changes is not related to large carnivores. Many changes also took place before the reintroduction of the carnivores and the creation of the CMZs.

The differences between areas inside CMZs and areas outside CMZs are negligible in terms of development in number of farmers and farmland. The main difference is related to sheep farming and is an addition to the structural changes suffered by the agricultural sector in general. While the number of sheep declined slightly in the CMZs during the study period, the reduction was offset by an increase outside the CMZs. The total production was therefore maintained, but sheep farming was lost as a source of income for many farmers in the CMZs. The change is piecemeal, engendering a gradual removal of livestock from the range of carnivores.

Moving entire communities away from areas prone to carnivore attacks has been part of the conservation strategy in some countries (Nyhus and Tilson 2010). The geographical shift observed in Norway is not of that kind, but can be interpreted as a continuous social and economic process. Similar slow geographical shifts in livestock production is found in many parts of the world, and can be caused by climatic (Williams et al. 2016), ecological (Anadon et al. 2014), industrial (Lundström 2011), political (Saizen et al. 2010), or economic (Harrington et al. 2010) changes. The systematic geographical shift away from the CMZs in Norway is limited to sheep production, and carnivores or the carnivore management system is assumed to be a contributing factor.

Limited, occasional and evenly distributed losses are bearable for farmers, especially if they are sufficiently compensated. It is, however, challenging to create and implement a fair and acceptable compensation scheme (Nyhus et al. 2005). The losses are not evenly distributed: a few farmers suffer large and repeated losses (Landa et al. 1999). Losses are demotivating to the farmers that are affected (Vittersø et al. 1998), and more so if the compensation is perceived as unfair. The Norwegian compensation scheme has been challenged in court and the court ruled that the administrative practice used to determine compensations was unpredictable (Frostating 2013). The emotional and economic effect of accumulated losses is that farmers who suffer large and repeated losses give up livestock agriculture. The vacancy in the market is subsequently filled by farmers in other parts of the country.

### Sheep Farming where CMZs Overlap

Sheep farming has declined sharply in areas where three or four CMZs overlap: these areas coincide with the CMZ for brown bear, where the relative reduction in the number of sheep is particularly high (−39%). It is not possible to separate the effects of having many CMZs in an area, and the effects of the management zone for brown bears alone. The CMZ for brown bear is always present where three or four CMZs overlap.

Bear attacks on grazing sheep has been prevalent in Norway (Mysterud 1980). Bears are large, unpredictable, and occasionally violent and therefore represent a threat that the farmers are unable to cope with. Bears can damage carnivore-repellant fences and the damage inflicted on a herd attacked by brown bear is often substantial with many animals killed. The CMZ for brown bear is found in regions where livestock farming is particularly dependent on using outfield resources. Bears are incompatible with free-roaming sheep in the outfields and prevent the farmers from exploiting these resources. Farms in the CMZ for brown bear are small and herds cannot be sustained on their infields alone. The result is that sheep farmers are forced out of business.

This development in areas where three or four CMZs overlap explains the geographical shift in sheep production as discussed above. It is not a general shift away from CMZs. The change is negligible in areas with only one or two CMZs. We interpret the change as a reaction from farmers suffering high losses and leaving the sheep industry in the most affected areas, with a corresponding increase in herd sizes outside the CMZs.

Cattle or dairy production could constitute a viable alternative for sheep farmers. Norwegian authorities have in some cases offered grants to farmers who are willing to change to dairy farming. Brown bear is, however, a danger to cattle as well as sheep. Cattle production also require higher investments than sheep farming. Farmers on small farms are less inclined to accept this financial risk. Cattle furthermore require access to more infield areas, so several sheep farmers have to go out of business before one of them can buy or rent the land from the rest in order to create a sufficiently large production unit for cattle or dairy production.

Carnivores are not the only possible explanation for the structural changes in the regions where CMZs overlap. An alternative explanation is that this is remote, rural areas that people (in particular young people) find socially unattractive. They therefore abandon farming and migrate to urban areas. This rural depopulation is an ongoing process in Western Europe, driven by socioeconomic factors (MacDonald et al. 2000; Rey-Benayas et al. 2007). According to this explanation, the areas where the CMZ for brown bear is located and three or four CMZs overlap will sink into deselation irrespective of the presence of carnivores. Further studies are needed to test this hypothesis by comparing the development in areas with overlapping CMZs to similar, remote areas with no CMZs.

### Differences Inside the CMZ for Gray Wolf

There are notable geographical differences in the development inside the CMZ for gray wolf (Table 1). The number of sheep was reduced by −42% in the northern part of the zone, but increased by 55% in the southern part. These apparently contradictory results are related to several factors. One factor is that the northern part coincides with the CMZ for brown bear, as well as wolverine and lynx. This is the only region in Norway falling inside the CMZ for all four large carnivores. Clearly, the pressure from the carnivores is larger in this area than in any other part of the country. The southern region is inside the CMZ for lynx, but bear and wolverine are not present. There is also more wolfs in the north than in the south. The impact of carnivores is therefore substantially lower in the south than further north.

Much of the southern part of the CMZ for gray wolf is within commuting distance from the capital Oslo and several other large cities. People who inherit small farms in this region can find employment in nearby urban centers. Some city dwellers also find it attractive to move out of the cities and settle on small farms in commuting distance from the urban areas. These rural residents earn their main income outside the agricultural sector, but often keep horses and a few sheep on the farm.

Sheep in this region are kept in fenced enclosures on managed fields, patches of forest land, or in ravines close to the infields. Predator-repellent electric fences are common, and subsidized by the authorities. Losses occur here as well, but with little consequence for the farmer’s economy. Grants provided for fencing, compensation for inconveniences of keeping the sheep on the infields and grants for landscape management may be insufficient incitements for farmers who have sheep production as a main part of their income, but is an attractive subvention for farmers keeping small herds of sheep for landscape management or as an avocation.

### Outfield Grazing Resources are less Exploited in CMZs than Outside CMZs

A notable difference between the CMZs for carnivores and the rest of the country is the exploitation of the outfield grazing capacity. Norway has outfield grazing resources to feed ~7,500,000 LSU (1 LSU = 1 adult sheep) in the outfields during the summer season. The current use is ~3,000,000 LSU, or 40% of the capacity. The use outside the CMZs amounts to 59% of the capacity, while the use inside the CMZs for carnivores is only 26% of the capacity. The differences are even more striking when the CMZs are studied individually. Only 6% of the grazing resources in the CMZ for brown bears are used, while the use in the CMZ for gray wolf is 12%. It is reasonable to interpret these differences as an effect of the presence of carnivores and regulations implemented as part of the zoning strategy.

The low number of livestock in some of the carnivore areas will inevitably also lead to changes in the cultural landscape and influence the biodiversity in these areas. A number of studies describe how grazing livestock have formed the current vegetation and biodiversity in farmland and pastures in Norway (Vandvik and Birks 2002, 2004; Potthoff 2009; Wehn et al. 2011). The consequence of a declining number of livestock is that low grazing pressure leads to succession towards woodland (Olsson et al. 2004; Bryn et al. 2013; Speed et al. 2010; Wehn 2009) accompanied by a negative influence on the biodiversity (Johansen et al. 2019; Potthoff and Stroth 2011). Plants typical for seminatural meadows and pastures are replaced by plants that are less resistant to grazing when the livestock is gone (Speed et al. 2012).

A possible solution for some regions is to change the production from sheep to cattle, at least in areas without bears. Cattle could uphold the grazing intensity, in particular on areas close to the farms, and contribute ecosystem services by upholding biodiversity and landscape qualities. Use of traditional Norwegian races, now threatened by extinction, could also be a step to support the conservation of genetic variation in Norwegian agriculture (Sæther 2013). Sheep in fenced enclosures around the farmsteads contribute to uphold the landscape and biodiversity on these selected sites.

We observed a geographical difference with respect to the changes in area used for grass production. These areas were reduced by −3.3% outside the CMZs, but increased by 5.1% inside the CMZs. The increase in the southern part of the CMZ for gray wolf was 16.7%. We interpret the differences as a sign of increasing reliance on infield pasture and fodder produced on the farm itself, at the expense of outfield pastures in areas where the livestock is vulnerable to carnivore attacks. Farmers have, for example, established community-based systems to handle situations when carnivores, such as the wolverine, habitually start their attacks on livestock late in summer (Mabille et al. 2015). Sheep are mustered and brought down from outfield pasture in the mountains to be kept on lowland pastures closer to the farms when the carnivore attacks set in. This increases the demand for cultivated fodder.

### Carcasses of Sheep Killed by Carnivores are Mainly Found near, but Outside the CMZs

Sheep losses have decreased inside the CMZs since the implementation of the zoning strategy. The decrease is most noticeable inside the CMZs for brown bear and gray wolf. This is an effect of the removal of sheep from the open outfield pastures. The physical separation of livestock and carnivores is effective. The liability is increased losses in areas just outside the CMZs and that some farmers lose their employment.

The borders of the CMZs are not clear to the animals, except when they follow large water bodies. Roaming or migrating carnivores will not heed zonal borders. Resident carnivores in the CMZs will also stray outside the zones. This is particularly true for dispersing bears and wolves searching for new territories (Swenson et al. 1998; Linnell et al. 2005a; Kojola et al. 2006; Ministry of Climate and Environment 2016, p. 26). While CMZs are filling up with established family groups as a result of successful conservation strategies, more and more individuals also straggle outside the zones. Our results show that a substantial part of the depredation by carnivores takes place outside the CMZ borders. Similar effects were observed in Italy where the highest level of conflict was found at the border of the wolf range (Ciucci and Boitani 1998). This is an unforeseen effect of the zoning strategy. More resolute hunting of carnivores straying outside the CMZs is needed to relieve the problem, and farmers in the neighborhood outside a CMZ will need some of the same protective measures that are used inside the CMZs.

### Mitigation Efforts

The reintroduction of carnivores in Norway has led to depredation on livestock, but the losses do not represent a threat to national food security and has little impact on the economy of the agricultural sector at large. The consequences can still be considerable for the economy and quality of life for individual farmers. This is in accordance with the results reported by Rigg et al. (2011) from their study of human-livestock conflicts in Slovakia. The reintroduction of carnivores is consequently controversial and leads to conflicts. Zoning is a mitigation strategy aiming to minimize these conflicts. The actions involved are (1) to manipulate large carnivore density; (2) to adjust the way conflicting activities are conducted; and (3) to remove conflicting activity from the carnivore range (Linnell et al. 2005b).

Carnivore density is regulated by lethal control. Zoning implies that carnivore populations must be culled in order to control their size and geographical distribution. Due to the twofold objective of the Norwegian zoning policy, carnivores must be removed when they are a threat to livestock outside the CMZs. Our results show that this aspect of the zoning strategy is unsuccessful. Sheep losses are high in areas outside, but close to the CMZs. The problem is particularly severe in the vicinity of the CMZs for gray wolf and brown bear. The challenge is partly that hunting during acute situations is difficult, especially in forested areas during the summer, but also that environmental authorities may be reluctant to permit culling as an emergency measure. Better, more efficient hunting outside the CMZs is needed to strengthen the legitimacy of the zoning strategy.

The CMZs are not wildlife reserves, but created to give carnivores a place to breed in order to reach a viable population size. The population must still be controlled upon reaching the preset population size. Culling inside the zones is required to limit the spillover effect caused by carnivores migrating outside the zones and to create vacant spaces inside the zones where vagrants can settle and create a territory.

Removal of packs or individuals can reduce conflict by creating an interruption to the local carnivore pressure, not only for sheep farmers but also for local hunters. Culling inside the CMZs does also have a direct effect on conflicts when local hunters are allowed to participate. “Norwegian studies leave little doubt that one of the measures that potentially could have the greatest conflict-reducing effect is carnivore hunting in a form that is open to local hunters” (Linnell et al. 2005b, p. 173).

The second action involved in the zoning management is adjustment in the way conflicting activities are conducted. This is by applying restrictions as well as through stimulation. Restrictions include legislation forbidding livestock on open (unfenced) pasture and limiting credits for investments in sheep industry in carnivore prone areas. Financial incentives include grants for fencing, keeping sheep in enclosures, and coverage for expenses induced by the structural changes in the sheep industry. The efforts are, however, not always successful. Two very large enclosures have been established in the northern part of the CMZ for gray wolf. Lynx and brown bear have both been able to enter these enclosures, clearly with the potential to create much carnage. Hunting carnivores inside the enclosures is difficult due to their large acreage. The learning point is that smaller enclosures are more beneficial, because they are easier to guard and also less costly to maintain. Smaller enclosures have been prioritized in the southern part of the CMZ for gray wolf. The downside of smaller enclosures is that sheep must be moved between enclosures more often, and that higher sheep density leads to more medical problems (Lillevold 2015). Still, smaller enclosures are preferable but the grants have to be sufficient to cover the real cost of setting up and maintaining the fences, moving the sheep between enclosures, cover medical expenses, and offset the reduced production when sheep graze in enclosures. The economical aspect of keeping sheep on infield pasture for prolonged periods is documented in Stornes (2017).

The third aspect of zoning is to remove conflicting activity from the carnivore range. This can be done by using shepherds and guarding dogs, or by confining livestock to fenced pastures (Linnell et al. 2012). Shepherding and guarding dogs have been successful in other countries, but does not give results in Norway (Mabille et al. 2015). This is partly due to the behavior of the sheep races used in Norway, partly linked to the terrain and vegetation.

Forced migration is used in some countries, but not in Norway. Still, the sheep industry is effacing from some regions within the CMZs. This is partly achieved by grants supporting the change from sheep farming to other productions (Jenssen et al. 2019), but mainly happening because farmers find that the losses and cost is too high and therefore choose to leave the industry. This process is generating conflict (Vittersø et al. 1998).

The process leading to complete abandonment is long and painful. Farmers interviewed in Strand et al. (2018) found the process alienating, impairing confidence in the authorities, and generating conflict. The anger is not only directed at the carnivores, but also against the environmental authorities. The observation is concordant with reports from Sweden (Eriksson 2017).

The consequence of zoning when the largest carnivores (brown bear and gray wolf) are involved is that many farmers abolish the entire industry. The abatement has been a prolonged process and this is itself generating conflict (Strand et al. 2018). The management of the implementation of the zoning strategy is therefore important.

The prospect that the traditional sheep industry based on outfield resources would be abolished in the most carnivore prone areas could have been communicated clearly to the affected farmers when the CMZs were established. This would not have been a popular message, but it would have allowed the farmers and the local communities to be better prepared for the inevitable changes. Sufficient funding could have been allocated to allow farmers to change to other kinds of agriculture without suffering economic losses. Grants could also have been given to farmers who wanted to retrain themselves for other vocations or develop nonagricultural businesses based on the farms. The authorities could also have created alternative employment opportunities in the rural areas. Farms in peri-urban areas are upheld as residences because the owner can find alternative employment. Farmers forced out of business in more remote areas do not have the same opportunities.

The zoning policy also has consequences outside the agricultural sector. Skogen (2001) observed that opposition to the current carnivore policy often comes from people not themselves involved in sheep farming. Some of these are forest owners who find that their income from game hunting vanishes (Strand et al. 2016). Economic compensation to forest owners to cover lost income could lessen the conflict in areas with gray wolf. Engaging local hunters for monitoring (Skogen 2003), culling (Linnell et al. 2005b), and otherwise controlling carnivore populations would also improve local involvement and could lessen the conflicts.

## Conclusion

Our results show that the zonal management approach used in Norway is a reasonably successful management strategy when the goal is to separate livestock from carnivores and reduce the livestock losses. The strategy does, however, imply an unequal distribution of burdens. The encumbrance is severe for livestock farmers inside the CMZs, who use time and resources to implement mitigation measures. Carnivore presence and the increased use of (fenced) infield pastures is causing reduced animal welfare and increasing medical costs (Asheim and Eik 2005; Kilgour et al. 2008). Farmers in the most affected areas cannot use their local grazing resources in the outfields and many have abandoned livestock farming altogether, with considerable consequences for the economy and the quality of life for the farmers concerned and their local communities. There are few attempts to seriously involve local communities in the management of the zoning strategy, compensation for incommodities is deficient and there are insufficient alternative employment opportunities for affected farmers. The legitimacy of the CMZ management system is therefore disputed in many rural communities, escalating the political conflict over the entire carnivore conservation strategy.

Economic compensation to cover the burden imposed by the carnivore policy may relieve the conflict. For sheep farmers, the reasonable compensation should cover the cost needed to secure an income equal to the income from sheep farming. Policy makers could also consider allowing former farmers a freedom of choice between transformation to a new kind of agricultural production (e.g., dairy farming), continued sheep farming under new and confined conditions (e.g., on fenced infields), or finding alternative employment opportunities. Compensation for forest owners who had their income from game hunting reduced could also be considered, if the aim is to reduce the human–carnivore conflict. These proposals are in effect hypotheses about their assumed positive effect as means to reduce human–carnivore conflicts. It is difficult to test these hypotheses effectively, unless the policy is implemented, but it is possible to conduct studies of attitudes towards the proposals.

There is a need for further monitoring and research, starting with the documentation of the carnivore management system itself. The available maps of the CMZs used in this study required considerable technical management before they could be used in the analysis. It was not possible to find maps of the carnivore distribution and we had to develop an approximation for use in this study.

Our results show that the major structural change in sheep farming is found in areas where three or four CMZs overlap. This is indicative of a causal relationship with carnivores, but the exact relationship cannot be determined without better data documenting carnivore presence and density. Such data are needed to separate the effect of bears alone from the effect of having many carnivore individuals or many carnivore species together in an area.

We assume that the zoning strategy can have an impact on biodiversity and the agricultural landscape (negative in areas where livestock is removed and positive at locations where livestock is grazing more intensively in fenced enclosures). No monitoring data are currently available to test this hypothesis. The register data used here also show that livestock farming is abandoned in remote rural areas inside the CMZs. This may be an effect of the carnivore management, but could also be a consequence of migration from rural to urban areas, independent of the presence of carnivores. A comparative study with similar regions outside the CMZs is needed in order to test this hypothesis.

Our results show that sheep losses are increasing outside, but close to the CMZs. The range probably varies between carnivores and possibly also with terrain and vegetation. A better understanding of the distance factor would be beneficial in order to design management zones with less impact on livestock outside the borders. More knowledge is also needed to design and implement geographically accurate mitigation efforts to assist farmers vulnerable to predation on livestock outside the CMZs.

Finally, there are many studies of people’s attitude towards carnivores (Dressel et al. 2014; Krange et al. 2017). There are, however, few studies of the wider social and economic consequences of zonal carnivore management systems on local communities and people’s economy and welfare. Skogen (2001) and Skogen and Krange (2003) show that conflict over carnivore management reach beyond the sheep farmers who are directly affected. We believe conflict management must start with a thorough understanding of the social aspects of the conflict. Currently, there is a knowledge gap regarding the social consequences of carnivore management on rural societies.

## References

[CR1] Anadon JD, Sala OE, Maestre FT (2014). Climate change will increase savannas at the expense of forests and treeless vegetation in tropical and subtropical Americas. J Ecol.

[CR2] Asheim LJ, Mysterud I (2004). Economic impact of protected large carnivores on sheep farming in Norway. Sheep Goat Res J.

[CR3] Asheim LJ, Eik LO (2005). Animal welfare conditions for free ranging sheep in Norwegain predator habitats. Biotechnol Anim Husb.

[CR4] Baker PJ, Boitani L, Harris S, Saunders G, White PL (2008). Terrestrial carnivores and human food production: impact and management. Mammal Rev.

[CR5] Behmanesh M, Malekian M, Hemami MR, Fakheran S (2019). Patterns and determinants of human–carnivore conflicts in Central Iran: realities and perceptions behind the conflict. Hum Dimens Wildl.

[CR6] Blekesaune A, Rønningen K (2010). Bears and fears: cultural capital, geography and attitudes towards large carnivores in Norway. Nor J Geogr.

[CR7] Bryn A, Dourojeanni P, Hemsing LØ, O’Donnell S (2013). A high-resolution GIS null model of potential forest expansion following land use changes in Norway. Scand J For Res.

[CR8] Bryn A, Strand GH, Angeloff M, Rekdal Y (2018). Land cover in Norway based on an area frame survey of vegetation types. Nor J Geogr.

[CR9] Bye AS, Aarstad PA, Løvberget AI, Høie H (2014). Jordbruk og miljø, Tilstand og utvikling 2013, SSB Report 2014/10. Statistics Norway, Oslo (in Norwegian)

[CR10] Cardillo M, Purvis A, Sechrest W, Gittleman JL, Bielby J, Mace GM (2004). Human population density and extinction risk in the world’s carnivores. PLoS Biol.

[CR11] Chapron G, Kaczensky P, Linnell JDC, von Arx M, Huber D, Andrén H, López-Bao JV, Adamec M, Álvares F, Anders O, Balčiauskas L, Balys V, Bedő P, Bego F, Blanco JC, Breitenmoser U, Brøseth H, Bufka L, Bunikyte R, Ciucci P, Dutsov A, Engleder T, Fuxjäger C, Groff C, Holmala K, Hoxha B, Iliopoulos Y, Ionescu O, Jeremić J, Jerina K, Kluth G, Knauer F, Kojola I, Kos I, Krofel M, Kubala J, Kunovac S, Kusak J, Kutal M, Liberg O, Majić A, Männil P, Manz R, Marboutin E, Marucco F, Melovski D, Mersini K, Mertzanis Y, Mysłajek RW, Nowak S, Odden J, Ozolins J, Palomero G, Paunović M, Persson J, Potočnik H, Quenette P-Y, Rauer G, Reinhardt I, Rigg R, Ryser A, Salvatori V, Skrbinšek T, Stojanov A, Swenson JE, Szemethy L, Trajçe A, Tsingarska-Sedefcheva E, Váňa M, Veeroja R, Wabakken P, Wölfl M, Wölfl S, Zimmermann F, Zlatanova D, Boitani L (2014). Recovery of large carnivores in Europe’s modern human-dominated landscapes. Science.

[CR12] Ciucci P, Boitani L (1998). Wolf and dog depredation on livestock in Central Italy. Wildl Soc Bull.

[CR13] Dorresteijn I, Hanspach J, Kecskés A, Latkova H, Mezey Z, Sugár S, von Wehrden H, Fischer J (2013). Human-carnivore coexistence in a traditional rural landscape. Landsc Ecol.

[CR14] Dressel S, Sandström C, Ericsson G (2014). A meta‐analysis of studies on attitudes toward bears and wolves across Europe 1976–2012. Conserv Biol.

[CR15] Eriksson M, Sandström C, Ericsson G (2015). Direct experience and attitude change towards bears and wolves. Wildl Biol.

[CR16] Eriksson M (2017). Political alienation, rurality and the symbolic role of Swedish wolf policy. Soc Nat Resour.

[CR17] Forbord M, Bjørkhaug H, Burton RJF (2014). Drivers of change in Norwegian agricultural land control and the emergence of rental farming. J Rural Stud.

[CR18] Frostating (2013) Court ruling 13-041160ASD-FROS, Frostating Lagmannsrett, Trondheim (in Norwegian)

[CR19] Gervasi V, Linnell JD, Brøseth H, Gimenez O (2019) Failure to coordinate management in transboundary populations hinders the achievement of national management goals: the case of wolverines in Scandinavia. J Appl Ecol. 10.1111/1365-2664.13379

[CR20] Hansen I, Strand GH, de Boon A, Sandström C (2019) Impacts of the Norwegian large carnivore management strategy on the national grazing sector. J Mt Sci. 10.1007/s11629-019-5419-6

[CR21] Harrington LM, Lu M, Kromm DE (2010). Milking the plains: movement of large dairy operations into Southwestern Kansas. Geographical Rev.

[CR23] Jenssen E, Landrø A, Stornes OK, Vasseljen J, Ystad E, Uggen KT (2019) Omstilling fra saueproduksjon grunnet rovvilttap og ‐skader. Vurderingsgrunnlag for beregning av tilskuddssatser, NIBIO Report 64/2019, Ås (in Norwegian), p 24

[CR83] Johansen Line, Taugourdeau Simon, Hovstad Knut Anders, Wehn Sølvi (2019). Ceased grazing management changes the ecosystem services of semi-natural grasslands. Ecosystems and People.

[CR24] Kaltenborn BP, Bjerke T (2002). The relationship of general life values to attitudes toward large carnivores. Hum Ecol Rev.

[CR25] Kaartinen S, Luoto M, Kojola I (2009). Carnivore-livestock conflicts: determinants of wolf (Canis lupus) depredation on sheep farms in Finland. Biodivers Conserv.

[CR26] Kilgour R, Waterhouse T, Dwyer C, Ivanov I (2008) Farming systems for sheep production and their effect on welfare. In: Dwyer CM (ed) The welfare of sheep, animal welfare, vol 6. Springer, Dordrecht, 10.1007/978-1-4020-8553-6_6

[CR27] Kojola I, Aspi J, Hakala A, Heikkinen S, Ilmoni C, Ronkainen S (2006). Dispersal in an expanding wolf population in Finland. J Mammal.

[CR28] Krange O, Odden J, Skogen K, Linnell JDC, Stokland HB, Vang S, Mattisson J (2016). Evaluering av regional rovviltforvaltning, NINA Report 1268.

[CR29] Krange O, Sandström C, Tangeland T, Ericsson G (2017). Approval of wolves in Scandinavia: a comparison between Norway and Sweden. Soc Nat Resour.

[CR30] Landa A, Gudvangen K, Swenson JE, Røskaft E (1999). Factors associated with wolverine Gulo gulo predation on domestic sheep. J Appl Ecol.

[CR31] Lillevold HG (2015) Evaluating the opportunities and barriers to implementing changes in sheep farming practices in Norway. MSc thesis in Natural Resources Management, Department of Geography, Norwegian University of Science and Technology, Trondheim. http://hdl.handle.net/11250/2448275

[CR32] Linnell JDC, Brøseth H, Solberg EJ, Brainerd SM (2005). The origins of the southern Scandinavian wolf Canis lupus population: potential for natural immigration in relation to dispersal distances, geography and Baltic ice. Wildl Biol.

[CR33] Linnell JDC, Nilsen EB, Lande US, Herfindal I, Odden J, Skogen K, Andersen R, Breitenmoser U (2005b) Zoning as a means of mitigating conflicts with large carnivores: principles an reality, In: Woodroffe, R, Thirgood, S, Rabinowitz, A (eds) People and wildlife: conflict or coexistence No 9, Cambridge University Press, Cambridge, pp. 162–175

[CR34] Linnell John D. C., Odden John, Mertens Annette (2012). Mitigation methods for conflicts associated with carnivore depredation on livestock. Carnivore Ecology and Conservation.

[CR35] López-Bao JV, Aronsson M, Linnell JDC, Odden J, Persson J, Andrén H (2019). Eurasian lynx fitness shows little variation across Scandinavian human-dominated landscapes. Sci Rep.

[CR36] Lundström M (2011). Dynamics of the livestock revolution: marginalization and resistance in southern brazil. J Sustain agriculture.

[CR37] Mabille G, Stien A, Tveraa T, Mysterud A, Brøseth H, Linnell JDC (2015). Sheep farming and large carnivores: what are the factors influencing claimed losses?. Ecosphere.

[CR38] MacDonald D, Crabtree JR, Wiesinger G, Dax T, Stamou N, Fleury P, Gutierrez Lazpita J, Gibon A (2000). Agricultural abandonment in mountain areas of Europe: environmental consequences and policy response. J Environ Manag.

[CR39] Martin MA (2001). The future of the world food system. Outlook Agric.

[CR40] Mattiello S, Brescani T, Gaggero S, Russo C, Mazzarone V (2012). Sheep predation: characteristics and risk factors. Small Rumin Res.

[CR41] Meuret M, Garde L, Moulin CH, Nozières-Petit MO, Vincent. M (2017). Élevage et loups en France: historique, bilan et pistes de solution, vol 30.

[CR42] Ministry of Environment (2003) Rovvilt i norsk natur, St.meld. nr. 15 (2003–2004). Government white paper, in Norwegan, Oslo. https://www.regjeringen.no/no/dokumenter/stmeld-nr-15-2003-2004-/id403693/

[CR43] Ministry of Climate and Environment (2016) Ulv i norsk natur, Bestandsmål for ulv og ulvesone. Government white paper 21 (2015-2016), Oslo, https://www.regjeringen.no/no/dokumenter/meld.-st.-21-20152016/id2480008/

[CR44] Mysterud Ivar (1980). Bear Management and Sheep Husbandry in Norway, with a Discussion of Predatory Behavior Significant for Evaluation of Livestock Losses. Bears: Their Biology and Management.

[CR45] Navarro Laetitia M., Pereira Henrique M. (2015). Rewilding Abandoned Landscapes in Europe. Rewilding European Landscapes.

[CR46] Nyhus PJ, Osofsky SA, Ferraro P, Madden F, Fischer H, Woodroffe R, Thirgood S, Rabinowitz A (2005). Bearing the costs of human-wildlife conflict: the challenges of compensation schemes. People and Wildlife, Conflict Or Co-existence?, Conservation Biology Series 9.

[CR47] Nyhus Philip J., Tilson Ronald (2010). Panthera tigris vs homo sapiens. Tigers of the World.

[CR48] Olsson EGA, Hanssen S, Rønningen K (2004). Different conservation values of biological diversity? A case study from the Jotunheimen mountain range, Norway. Nor Geografisk Tidsskr-Nor J Geogr.

[CR49] Potthoff K (2009). Grazing history affects the tree-line ecotone: a case study from Hardanger, Western Norway. Fenn Int J Geogr.

[CR50] Potthoff K, Stroth V (2011). Patterns of vegetation change on alpine mountain summer farms in Norway. Geogr Ann Ser A Phys Geogr.

[CR51] Rey-Benayas JM, Martins A, Nicolau JM, Schulz JJ (2007). Abandonment of agricultural land: an overview of drivers and consequences. CAB Rev.

[CR52] Rigg R, Findo S, Wechselberger M, Gorman ML, Sillero-Zubiri C, MacDonald DW (2011). Mitigating carnivore–livestock conflict in Europe: lessons from Slovakia. Oryx.

[CR53] Røskaft E, Händel B, Bjerke T, Kaltenborn BP (2007). Human attitudes towards large carnivores in Norway. Wildl Biol.

[CR54] Safi K, Pettorelli N (2010). Phylogenetic, spatial and environmental components of extinction risk in carnivores. Glob Ecol Biogeogr.

[CR55] Saizen I, Maekawa A, Yamamura N (2010). Spatial analysis of time-series changes in livestock distribution by detection of local spatial associations in Mongolia. Appl Geogr.

[CR56] Scasta JD, Windh JL, Stam B (2018). Modeling large carnivore and ranch attribute effects on livestock predation and nonlethal losses. Rangel Ecol Manag.

[CR57] Skogen K (2001). Who’s afraid of the big, bad wolf? Young people’s responses to the conflicts over large carnivores in Eastern Norway. Rural Sociol.

[CR58] Skogen K (2003). Adapting adaptive management to a cultural understanding of land use conflicts. Soc Nat Resour.

[CR59] Skogen K, Krange O (2003). A wolf at the gate: the anti‐carnivore alliance and the symbolic construction of community. Sociologia ruralis.

[CR60] Skåtan JE, Lorentzen M (2011) Drept av rovvilt? Statens naturoppsyn. Miljødirektoratet, M-1319, Trondheim. https://www.miljodirektoratet.no/publikasjoner/2019/mars-2019/drept-av-rovvilt/

[CR61] Speed JDM, Austrheim G, Hester AJ, Mysterud A (2010). Experimental evidence for herbivore limitation of the treeline. Ecology.

[CR62] Speed JD, Austrheim G, Hester AJ, Mystrerud A (2012). Elevational advance of alpine plant communities is buffered by herbivory. J Vegetation Sci.

[CR63] Stahl P, Vandel JM, Herrenschmidt V, Migot P (2001). Predation on livestock by an expanding reintroduced lynx population: long‐term trend and spatial variability. J Appl Ecol.

[CR64] Stahl P, Vandel JM, Ruette S, Coat L, Coat Y, Balestra. L (2002). Factors affecting lynx predation on sheep in the French Jura. J Appl Ecol.

[CR65] Stornes OK (2017) Tidlig nedsanking av sau og bare innmarksbeite. Sats per dyr og dag ved mer innmarksbeite, NIBIO Report 100/2017, NIBIO, Ås (in Norwegian), p 31

[CR66] Strand GH (2013). The Norwegian area frame survey of land cover and outfield land resources. Nor Geografisk Tidsskr Nor J Geogr.

[CR67] Strand GH, Bloch VVH (2009). Statistical grids for Norway. Documentation of national grids for analysis and visualisation of spatial data in Norway. Document 2009/9.

[CR68] Strand GH, Rekdal Y, Stornes OK, Hansen I, Rødven R, Bjørn TA, Eilertsen SM, Haugen FA, Hovstad KA, Johansen L, Mathiesen HF, Rustad LJ, Svalheim E, When S (2016) Tovviltbestandenes betydning for landbruk og matproduksjon basert på norske ressurser, NIBIO Report 63/2016, NIBIO, Ås (in Norwegian), p 128

[CR69] Strand GH, Hillestad ME, Kildahl K, Rekdal Y, Hansen I, Mathiesen HF, Stenbrenden M, Fjellhammer E, Angeloff M, Bunger A, Stokstad G (2018) Beitebruk i ulvesona, NIBIO Report 121/2018, NIBIO, Ås (in Norwegian), p 100

[CR70] Swenson JE, Sandegren F, Søderberg A (1998). Geographic expansion of an increasing brown bear population: evidence for presaturation dispersal. J Anim Ecol.

[CR71] Swenson Jon E., Schneider Michael, Zedrosser Andreas, Söderberg Arne, Franzén Robert, Kindberg Jonas (2017). Challenges of managing a European brown bear population; lessons from Sweden, 1943–2013. Wildlife Biology.

[CR72] Sæther NH (2013). Country report for the preparation of The Second Report on the State of the World’s Animal Genetic Resources, Agriculture, including sector-specific data contributing to The State of the World’s Biodiversity for Food and Agriculture—Norway.

[CR73] Treves A, Karanth KU (2003). Human–carnivore conflict and perspectives on carnivore management worldwide. Conserv Biol.

[CR74] Vandvik V, Birks HJB (2002). Partitioning floristic variance in Norwegian upland grasslands into within-site and between-site components: are the patterns determined by environment or by land-use?. Plant Ecol.

[CR75] Vandvik V, Birks HJB (2004). Mountain summer farms in Røldal, western Norway –; vegetation classification and patterns in species turnover and richness. Plant Ecol.

[CR76] Vittersø J, Kaltenborn BP, Bjerke T (1998). Attachment to livestock and attitudes toward large carnivores among sheep farmers in Norway. Anthrozoös.

[CR77] Wehn S (2009). A map-based method for exploring responses to different levels of grazing pressure at the landscape scale. Agriculture Ecosyst og Environ.

[CR78] Wehn S, Pedersen B, Hanssen SK (2011). A comparison of influences of cattle, goat, sheep og reindeer on vegetation changes in mountain cultural landscapes in Norway. Landsc Urban Plan.

[CR80] Widman M, Elofsson K (2018). Costs of livestock depredation by large carnivores in Sweden 2001 to 2013. Ecol Econ.

[CR81] Williams R, Scholtz MM, Neser FWC (2016). Geographical influence of heat stress on milk production of Holstein dairy cattle on pasture in South Africa under current and future climatic conditions. South Afr J Anim Sci.

[CR82] Woodroffe R (2000). Predators and people: using human densities to interpret declines of large carnivores. Anim Conserv.

